# Transferrin receptor facilitates TGF-*β* and BMP signaling activation to control craniofacial morphogenesis

**DOI:** 10.1038/cddis.2016.170

**Published:** 2016-06-30

**Authors:** R Lei, K Zhang, K Liu, X Shao, Z Ding, F Wang, Y Hong, M Zhu, H Li, H Li

**Affiliations:** 1 West China Developmental and Stem Cell Institute, West China Second Hospital, and State Key Laboratory of Biotherapy, West China Hospital, Sichuan University, Chengdu 610041, China; 2 SARITEX Center for Stem Cell Engineering Translational Medicine, Shanghai East Hospital, Tongji University School of Medicine, Shanghai 200123, China; 3 Shenzhen Key Laboratory for Molecular Biology of Neural Development, Laboratory of Developmental and Regenerative Biology, Institute of Biomedicine and Biotechnology, Shenzhen Institutes of Advanced Technology, Chinese Academy of Sciences, Shenzhen, Guangdong 518055, China; 4 Department of Nutrition, School of Public Health, Zhejiang University, 866 Yuhangtang Road, Hangzhou 310058, China; 5 Department of Cell Biology & Physiology, University of Pittsburgh School of Medicine, Pittsburgh, PA 15261, USA; 6 Nerdbio Inc., SIP Biobay, Jiangsu 215213, China

## Abstract

The Pierre Robin Sequence (PRS), consisting of cleft palate, glossoptosis and micrognathia, is a common human birth defect. However, how this abnormality occurs remains largely unknown. Here we report that neural crest cell (NCC)-specific knockout of transferrin receptor (Tfrc), a well known transferrin transporter protein, caused micrognathia, cleft palate, severe respiratory distress and inability to suckle in mice, which highly resemble human PRS. Histological and anatomical analysis revealed that the cleft palate is due to the failure of palatal shelves elevation that resulted from a retarded extension of Meckel's cartilage. Interestingly, *Tfrc* deletion dramatically suppressed both transforming growth factor-*β* (TGF-*β*) and bone morphogenetic protein (BMP) signaling in cranial NCCs-derived mandibular tissues, suggesting that Tfrc may act as a facilitator of these two signaling pathways during craniofacial morphogenesis. Together, our study uncovers an unknown function of Tfrc in craniofacial development and provides novel insight into the etiology of PRS.

Neural crest cells (NCCs) are highly pluripotent stem cell populations that arise from the neural folds and migrate extensively to different regions, where they differentiate into a broad range of cell types to build up various structures. The cranial NCCs (CNCCs) originate from posterior forebrain and posterior hindbrain and contribute to much of bones, cartilages, cranial nerves, muscles and connective tissues in face and neck.^1, 2, 3, 4^ During vertebrate craniofacial development, the specification, migration, proliferation and differentiation of CNCCs are tightly controlled by a spatial and temporal signaling network.^5^ Aberrations in any node of these signaling networks could result in craniofacial malformations, such as cleft lip, cleft palate and mandibular hypoplasia.

The Pierre Robin Sequence (PRS) is a human craniofacial malformation (OMIM: 261800) characterized by U-shaped cleft palate, glossoptosis (backward displacement of the tongue) and micrognathia (abnormally small mandible).^6, 7, 8^ PRS occurs with an incidence of 1 : 8500 to 1 : 14 000 in live births and results in severe respiratory distress and feeding difficulties in infants.^6, 9, 10^ Mutations in several genes, including *BMP2*, *SATB2*, *COL2A1*, *COL11A1*, *COL11A2*, *SOX9* and *KCNJ2*, have been identified in PRS.^11, 12, 13, 14, 15^ Mandibular growth retardation is considered to be the most possible cause for PRS-cleft palate.^7^ In mammals, Meckel's cartilage has a close relation to the mandibular development. During embryonic development, the extension of Meckel's cartilage within the mandibular prominence promotes the elongation of mandible, which subsequently assists the flattening of tongue and permits the elevation and followed fusion of palatal shelves.^7, 16^ Multiple genetic evidences have demonstrated that defective mandible and Meckel's cartilage development could lead to cleft palate.^7, 17, 18, 19, 20, 21, 22^

Transferrin receptor (Tfrc) is a homodimeric trans-membrane glycoprotein that binds to iron-bound transferrin and mediates the extracellular iron to be delivered into cells via clathrin-dependent endocytosis.^23, 24^ Although Tfrc is widely believed to be important for iron acquisition by all mammalian cells, its physiological function for mammal's development is poorly understood. Here, we report that Tfrc is necessary for embryonic craniofacial development. Mice lacking *Tfrc* in NCCs exhibit micrognathia, cleft palate, severe respiratory distress and inability to suckle, which highly resemble human PRS. Histological and anatomical analysis revealed that cleft palate results from the failure of palatal shelves elevation, furthermore the failed palatal shelves elevation is due to retarded extension of Meckel's cartilage that is essential for mandible elongation. Molecularly, the ablation of *Tfrc* in NCCs significantly suppressed transforming growth factor-*β* (TGF-*β*) and BMP signaling in NCCs-derived mandibular tissues. Based on these data, we propose that Tfrc is a facilitator of TGF-*β* and BMP signaling and Tfrc-mediated activation of these two signaling pathways is essential for craniofacial morphogenesis.

## Results

### *Tfrc* deletion in NCCs causes craniofacial defects

**Figure 1:**
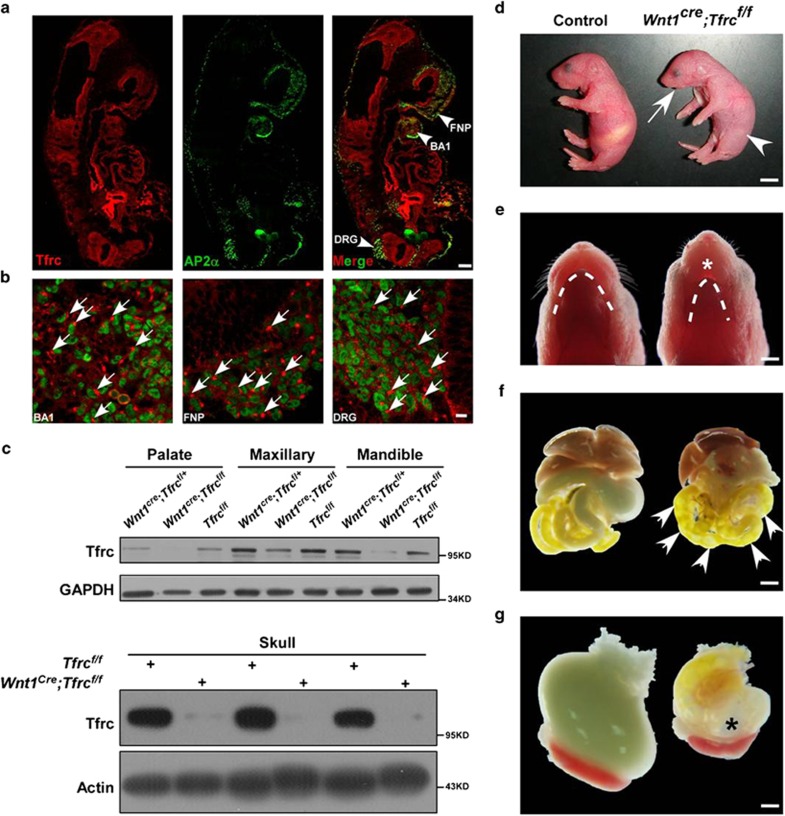
Conditional deletion of *Tfrc* in NCCs leads to mandible hypoplasia. (**a**) Expression pattern of Tfrc in NCCs-derived cells is detected by immunofluorescent staining of Tfrc (red) and AP-2*α* (green) in E9.5 embryo sections. (**b**) Magnification of the arrowheads indicated regions in **a**. Arrows mark the cluster of Tfrc in cell cytoplasm. (**c**) Expression of Tfrc protein in NCCs-derived palate, maxillary, mandible and skull. Tfrcs were reduced in *Wnt1*^*cre*^*;Tfrc*^*f/f*^ mutants compared with littermate controls. (**d**) Newborn *Wnt1*^*cre*^*;Tfrc*^*f/f*^ mutant exhibits shortened mandible (arrow) and no milk in stomach (arrowhead). (**e**) The ventral view of P0 *Wnt1*^*cre*^*;Tfrc*^*f/f*^mutant shows evident hypoplastic mandible (dotted line) and incomplete oral closure (asterisk). (**f**, **g**) P0 *Wnt1*^*cre*^*;Tfrc*^*f/f*^ mutants show abundant flatus in intestine (F, arrows) and stomach (**g**, asterisk). BA1, first branchial arch; BA2, second branchial arch; FNP, frontonasal prominence. Scale bar, 100*μ*m (**a**), 10*μ*m (**b**), 5 mm (**d**), 1.5 mm (**e**), 2.5 mm (**f**), 0.83 mm (**g**)

Although Tfrc has been studied extensively, the potential role of Tfrc in regulating NCCs development remains unclear. Therefore, we first examined the expression pattern of Tfrc during NCCs development by immunofluorescent staining. Sections from embryonic day 9.5 (E9.5) wild-type embryos were labeled with Tfrc and transcription factor activator protein 2*α* (AP-2*α*) antibodies, a marker for premigratory and migratory NCCs.^25, 26^ Tfrc was broadly and highly expressed in regions populated by cells of neural crest origin, including brachial arch (BA1), frontonasal prominence (FNP) and dorsal root ganglia (Figure 1a). In these regions, Tfrc showed a clustered expression pattern in cytoplasm (Figure 1b).

Besides its expression in NCCs-derived cells, Tfrc is expressed broadly during development, therefore it is not surprising that mice deficient for the Tfrc gene die before E12.5.^27^ To circumvent this early lethality and investigate the function of *Tfrc* in NCCs, we generated NCCs-specific *Tfrc* knockout (KO) mice by firstly crossbreeding *Wnt1*^*cre*^ mice^28^ with homozygous floxed *Tfrc* mice (*Tfrc*^flox/flox^)^29^ to get *Wnt1*^*cre*^*;Tfrc*^*f/+*^ heterozygote, then crossbreeding *Wnt1*^*cre*^*;Tfrc*^*f/+*^ heterozygote with *Tfrc*^flox/flox^ to get *Wnt1*^*cre*^*;Tfrc*^*f/f*^ mutants. The specificity and efficiency of *Tfrc* KO were examined by immunoblotting. As compared with control littermates, *Wnt1*-cre-mediated KO of *Tfrc* dramatically reduced Tfrc protein level in NCCs-derived craniofacial tissues, including palate, maxillary, mandible and skull (Figure 1c). *Wnt1*^*cre*^*;Tfrc*^*f/f*^ mutants were born at expected Mendelian ratios but all died within 24 h after birth. Morphologically, newborn *Wnt1*^*cre*^*;Tfrc*^*f/f*^ mutants exhibited micrognathia (Figures 1d and e), incomplete oral closure (Figure 1e), severe respiratory distress (Supplementary Material and Supplementary Movie 1) and inability to suckle, highly resembling the human PRS. Due to these severe defects, the mutant pups sucked air rather than milk into their gastrointestinal tract (Figures 1f and g). The morphologies of other tissues derived from NCCs were also examined in postnatal day 0 (P0) mutant pups. As shown, *Tfrc* KO in NCCs did not induce abnormalities in these tissues, including craniofacial nerves (Supplementary Figures S1A and B), dorsal root ganglions (Supplementary Figures S1 C–F) and cardiac development (Supplementary Figures S1 G–J). All these results suggest that *Tfrc* expression in NCCs is required for craniofacial development.

### *Wnt1*^*cre*^*;Tfrc*^*f/f*^ mutants exhibit cleft palate

**Figure 2:**
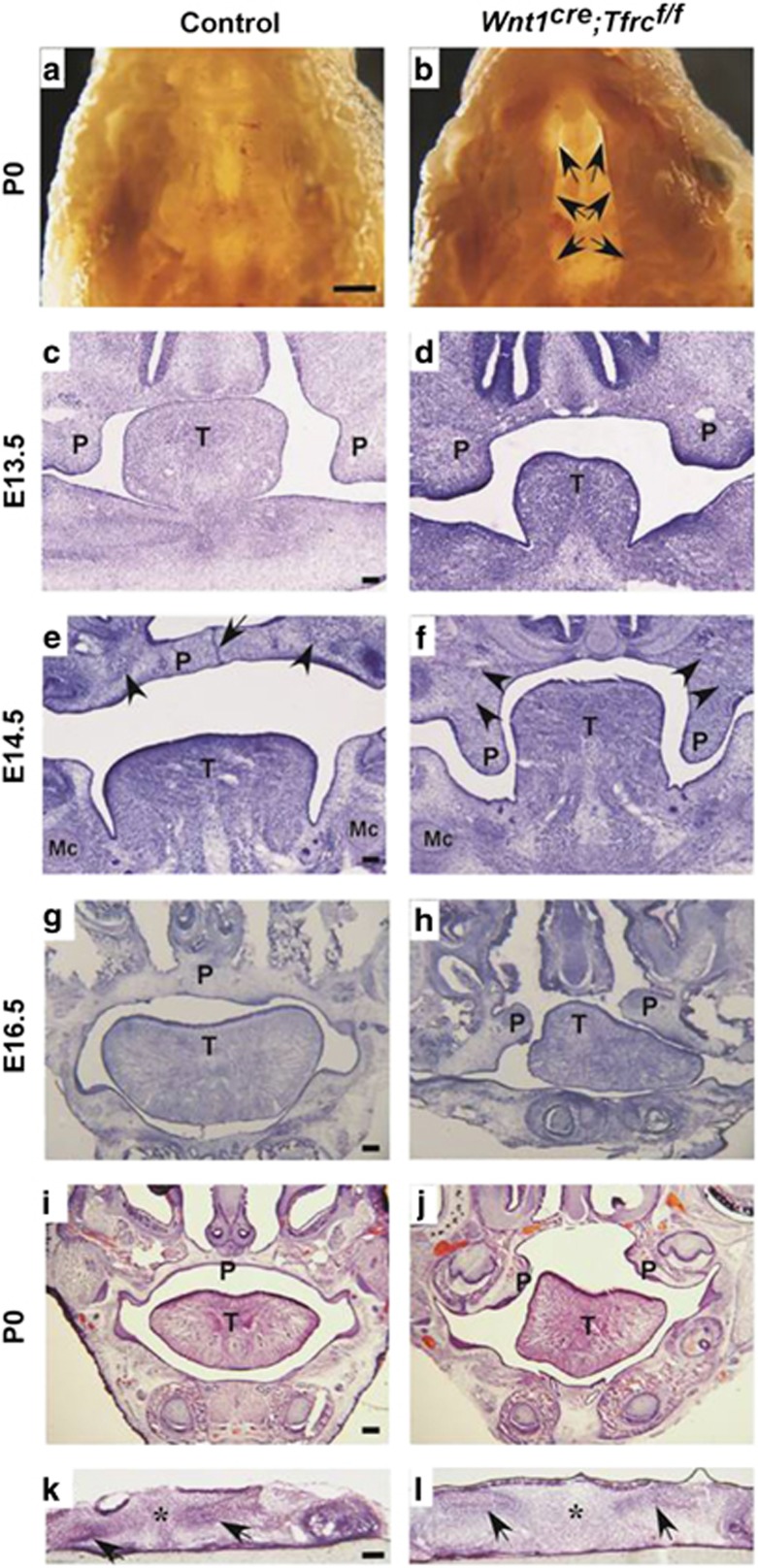
Conditional deletion of *Tfrc* in NCCs leads to cleft palate. (**a** and **b**) The ventral view of oral cavity shows fused palate in P0 control (**a**), but cleft palate in *Wnt1*^*cre*^*;Tfrc*^*f/f*^ mutant (**b**, arrows). (**c** and **d**) The palatal shelves in E13.5 *Wnt1*^*cre*^*;Tfrc*^*f/f*^ mutant and control both grow vertically at the two sides of tongue. (**e** and **f**) The palatal shelves in E14.5 control mouse have rotated horizontally above tongue and fused with remaining MEE (**e**, arrow), however, they fail to elevate and remain at the two sides of tongue in *Wnt1*^*cre*^*;Tfrc*^*f/f*^ mutant (**f**). Arrowheads in **e** and **f** mark cell aggregates formed normally in *Wnt1*^*cre*^*;Tfrc*^*f/*f^ mutant and control, which symbolize the precursors of palatal bone. (**g**–**j**) The palatal shelves in E16.5 and P0 controls have completely fused with disappearance of midline epithelium (**g** and **i**), in contrast to the cleft palates with arched tongues in *Wnt1*^*cre*^*;Tfrc*^*f/*f^ mutants (**h** and **j**). (**k** and **l**) *In vitro* organ culture shows palatal shelves in controls (*n*=17) and *Wnt1*^*cre*^*;Tfrc*^*f/f*^ mutants (*n*=7) all fused with complete disappearance of midline epithelium (asterisk), arrows indicate osteoid-like cell aggregated mass. P, palate; T, tongue; Mc, Meckel's cartilage. Scale bar, 1.5 mm (**a** and **b**), 0.1 mm (**c**–**l**)

Since phenotypes of respiratory distress and gastrointestinal tract filled with air bubbles are commonly seen in newborn mice with cleft palate, we removed mandible and observed a complete cleft palate in *Wnt1*^*cre*^*;Tfrc*^*f/f*^ mutant when compared with control littermate (Figures 2a and b). To determine the onset of cleft palate in mutants, the developments of palate at various embryonic stages were examined by histological staining. In E13.5 controls, the palatal shelves grew vertically down to the two sides of tongue (Figure 2c); then at E14.5, they rotated to a horizontal position above tongue and elongated to fuse with remnant medial edge epithelium (MEE) (Figure 2e), finally the E16.5 and P0 controls exhibited completely fused palate with flat tongues (Figures 2g and i). However, this process was disrupted by *Tfrc* deletion. The palatal shelves in E13.5 *Wnt1*^*cre*^*;Tfrc*^*f/f*^ mutants were well-developed and properly extended to the two sides of tongue (Figure 2d), but they failed to elevate above the tongue (Figure 2f), and consequently the E16.5 and P0 mutants displayed cleft palates with arched tongues (Figures 2h and j). The normal initiation of palatal bone were formed by mesenchymal cells both in *Wnt1*^*cre*^*;Tfrc*^*f/f*^ mutants and controls (Figures 2e and f), suggesting that Tfrc is not essential for palatal bone initiation but palatal fusion. Next, to examine the ability to fuse for palatal shelves *in vitro*, we performed the palatal shelves culture assay. In brief, palatal shelves were dissected from E13.5 embryos and placed in pairs on Millipore filters (EMD Millipore, Merck, KGaA, Darmstadt, Germany) with correct anterior–posterior orientation. By 3 days in culture, all these palatal shelves from *Wnt1*^*cre*^*;Tfrc*^*f/f*^ mutants and control littermates fused completely with normal disappearance of MEE (Figures 2k and l), suggesting that palatal shelves in *Wnt1*^*cre*^*;Tfrc*^*f/f*^ mutants retain the ability to fuse. In addition, formation of osteoid-like structures were observed in both control and mutant palatal mesenchyme (Figures 2k and l), indicating that the palatal bone initiation was not Tfrc dependent. Together, these results suggest that *Tfrc* depletion in NCCs leads to failed elevation of palatal shelves and thereby causes cleft palate.

### Craniofacial skeletons are malformed in *Wnt1*^*cre*^*;Tfrc*^*f/f*^ mutants

**Figure 3:**
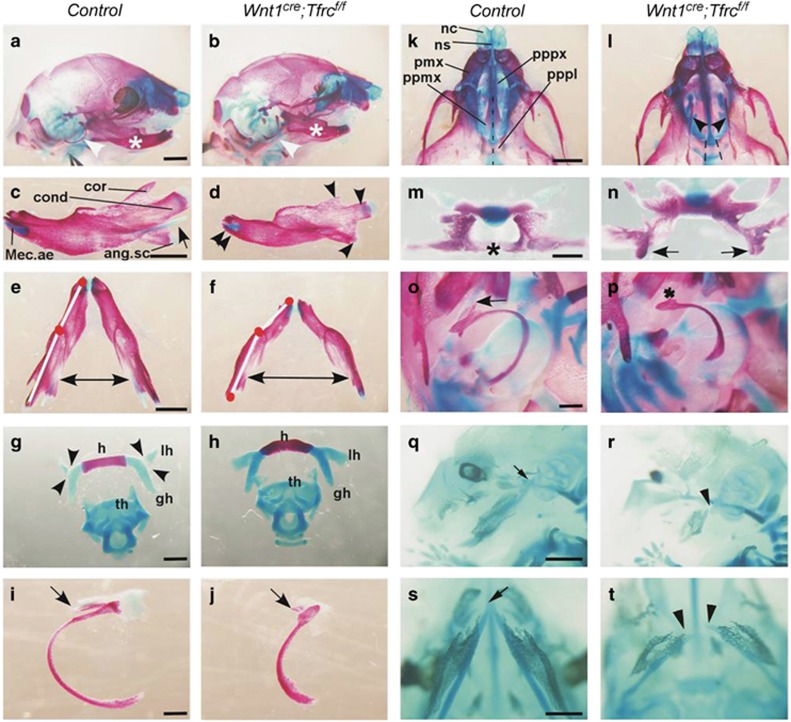
*Wnt1*^*cre*^*;Tfrc*^*f/f*^ mutants exhibit craniofacial skeleton defects. (**a** and **b**) Alizarin red and Alcian blue staining of bone and cartilage in P0 *Wnt1*^*cre*^*;Tfrc*^*f/f*^ mutant and littermate control. Asterisks and arrowheads indicate mandible and tympanic ring, respectively. (**c** and **d**) The lateral view of mandible dissected from neonates skeleton preparations. The extension of Meckel's cartilage is absent, while the distal symphysis formed normally (double arrowheads in **d**), but smaller size of mandibular bone (arrowheads in **d**) in *Wnt1*^*cre*^*;Tfrc*^*f/f*^ mutant. (**e** and **f**) The top view of mandible dissected from neonates skeleton preparations. Note the mis-angled mandibular bone (white line) and widened distance between mandibular bones (two-way arrow) in *Wnt1*^*cre*^*;Tfrc*^*f/f*^ mutant. (**g** and **h**) The ventral view of hyoid bone and laryngeal cartilages dissected from neonates skeleton preparations. Arrowheads in **g** indicate articulation between lesser horn and greater horn of hyoid bone in control, but lesser horn and hyoid bone are malformed in *Wnt1*^*cre*^*;Tfrc*^*f/f*^ mutant (**h**). (**i** and **j**) The lateral view of tympanic ring dissected from neonates skeleton preparations. Note the shortened tympanic ring and hypoplastic gonial bone (arrow in **j**) in *Wnt1*^*cre*^*;Tfrc*^*f/f*^ mutant. (**k** and **l**) The ventral view of skull from neonates skeleton preparations. Note the fusion of bilateral palatal bones in control (dotted line in **k**), but cleft in *Wnt1*^*cre*^*;Tfrc*^*f/f*^ mutant (arrowheads and dotted lines in **l**). (**m** and **n**) The horizontal view of palatal bones isolated from neonates skeleton preparations. Note the fusion of palatal bones in control (asterisk in **m**), but cleft with remnant of palatal bones in *Wnt1*^*cre*^*;Tfrc*^*f/f*^ mutant (arrows in **n**). (**o** and **p**) The lateral view of middle ear cartilages and tympanic ring dissected from neonates skeleton preparations. Note the Meckel's cartilage extends from distal to proximal otic capsule in control (arrow in **o**) but not in *Wnt1*^*cre*^*;Tfrc*^*f/f*^ mutant (asterisk in **p**). (**q** and **r**) Alcian blue staining of cartilage in E14.5 control and *Wnt1*^*cre*^*;Tfrc*^*f/f*^ mutant. Arrow in (**q**) indicates the proximal arms of Meckel's cartilage articulate normally with middle ear capsule in control, arrowhead in (**r**) indicates the failed extension of Meckel's cartilage to middle ear capsule in *Wnt1*^*cre*^*;Tfrc*^*f/f*^ mutant. Note the reduced extension of Meckel's cartilage in *Wnt1*^*cre*^*;Tfrc*^*f/f*^ mutant. (**s** and **t**) The ventral view of Meckel's cartilage in E14.5 embryos. Arrow in **s** indicates the fusion of Meckel's cartilages at distal tip, arrowheads in (**t**) indicate a gap between Meckel's cartilages in *Wnt1*^*cre*^*;Tfrc*^*f/f*^ mutant. Scale bar, 1mm (**a**–**f**, **q** and **r**), 3 mm (**g**–**j**), 1.5 mm (**k** and **l**), 4.5 mm (**m** and **n**), 2 mm (**o** and **p**, **s** and **t**). ang.sc, secondary cartilage of the angular process of mandible; cond, condylar process of the mandible; cor, coronoid process of the mandible; gh, greater horn of hyoid; h, hyoid; lh, lesser horn of hyoid; Mec.ae, anterior extremity of the Meckel's cartilage; nc, nasal capsule; ns, nasal septum; pmx, premaxilla; ppmx, palatal process of maxilla; pppl, palatal process of palatine; pppx, palatal process of premaxilla; th, thyroid cartilage

To examine the morphologies of craniofacial skeletons, whole-mount pups of *Wnt1*^*cre*^*;Tfrc*^*f/f*^ mutants and control littermates were stained with Alcian blue and Alizarin red to reveal cartilages and bones, respectively. In a line with the observed micrognathia in newborn mutant pups, the mandibular bones in *Wnt1*^*cre*^*;Tfrc*^*f/f*^ mutant were significantly shorter than that in control (Figures 3a and b). The secondary cartilage of angular process was nearly missing, while the mandibular condyle and coronoid process were also severely blocked/delayed by *Tfrc* deletion (Figures 3c and d). Moreover, the mandibular bones in *Wnt1*^*cre*^*;Tfrc*^*f/f*^ mutant were excessively curved, which consequently widened the distance between bilateral mandibular bones (Figures 3e and f). The hyoid bone (Figure 3h), tympanic ring and gonial bone (Figure 3j) were also deformed in *Wnt1*^*cre*^*;Tfrc*^*f/f*^ mutant when compared with that in control (Figures 3g and i). The cartilages of nasal capsule and gross morphologies of maxillary bones in *Wnt1*^*cre*^*;Tfrc*^*f/f*^ mutant and control were comparable (Figures 3k and l). The palatal process of palatine (pppl) and palatal process of maxilla (ppmx) extended horizontally and eventually fused to form the secondary palate (Figures 3k and m) in control. In contrast, these processes for secondary palate formation were disrupted in *Wnt1*^*cre*^*;Tfrc*^*f/f*^ mutant, both pppl and ppmx failed to elevate or fuse (Figures 3l and n).

NCCs-derived Meckel's cartilage is a significant determinant for mandibular development due to its inductive competence of initiating and regulating the ossification of mandible.^30^ To test whether the mandible defect was caused by abnormal development of Meckel's cartilage, we carefully analyzed the effect of *Tfrc* KO on developmental process of Meckel's cartilage. In contrast to that Meckel's cartilages were well-developed and extended from the symphysis of mandibular bones to malleus in P0 control (Figure 3o), no mature Meckel's cartilages were observed at the proximal end junction to malleus in *Wnt1*^*cre*^*;Tfrc*^*f/f*^ mutant (Figure 3p). Further examination showed that the defects of Meckel's cartilage occur as early as in E14.5 *Tfrc* KO embryos (Figures 3q–t). Alcian blue staining showed the well-developed Meckel's cartilage in control embryo (Figure 3q). In contrast, Meckel's cartilages were obviously smaller and the proximal arms of Meckel's cartilage were not articulated with middle ear capsule in *Wnt1*^*cre*^*;Tfrc*^*f/f*^ mutant (Figure 3r). Moreover, the Meckel's cartilages in mutant were not fused at the distal tip when compared with that in control (Figures 3s and t). These results indicate that Tfrc is essential for Meckel's cartilage maturation during embryonic development. Presumably, loss of *Tfrc* in NCCs primarily cause less developed Meckel's cartilage, consequently leading to mandible defect. Craniofacial skeletal abnormalities in *Wnt1*^*cre*^*;Tfrc*^*f/f*^ mutants were summarized in Supplementary Material Supplementary Table S1.

### Cleft palate in *Wnt1*^*cre*^*;Tfrc*^*f/f*^ mutant is secondary to mandibular defects

It has been documented that normal mandibular development is required for palate formation. Extension of Meckel's cartilage promotes the forward growth and elongation of lower jaw, which allows the reposition of tongue from its arched pattern to a flatten position in oral cavity and permits the subsequent palatal shelves elevation and fusion.^7, 16^ Given that the cleft palate phenotype in *Wnt1*^*cre*^*;Tfrc*^*f/f*^ mutant was tightly accompanied by shortened mandible and less matured Meckel's cartilage, we wondered whether the cleft palate is a secondary effect resulting from mandibular defects. To address this question, we employed *Nestin-cre* mediated *Tfrc* KO mice (*Nestin*^*cre*^*;Tfrc*^*f/f*^). *Wnt1*-cre-driven deletion of *Tfrc* occurs both in mandible and palatal mesenchyme, *Nestin-cre*-mediated *Tfrc* ablation happens in the mesenchyme and epithelium of palatal shelves but not in mandible.^31^ We observed that the newborn *Nestin*^*cre*^*;Tfrc*^*f/f*^ mutant does not phenocopy the *Wnt1*^*cre*^*;Tfrc*^*f/f*^ mutant but displays normal palatogenesis (Supplementary Figure S2). This result suggests that cleft palate in *Wnt1*^*cre*^*;Tfrc*^*f/f*^ mutant is not caused by intrinsic defects within palate but most probably is a secondary defect resulting from mandibular defects.

### NCC-specific deletion of *Tfrc* does not affect craniofacial NCCs migration or proliferation

**Figure 4:**
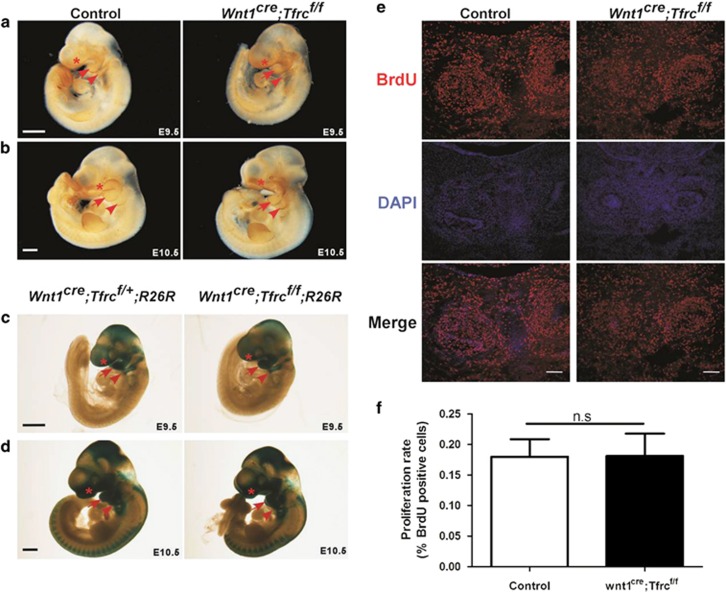
Conditional deletion of *Tfrc* in NCCs does not affect neural crest migration or cell proliferation. (**a** and **b**) Whole-mount AP-2*α* staining shows migratory neural crest cells in FNP (asterisk), BA1 (arrow) and BA2 (arrowhead). (**c** and **d**) Whole-mount X-gal staining shows neural crest cells in FNP (asterisk), BA1 (arrow) and BA2 (arrowhead). (**e** and **f**) Immunofluorescent labeling (**e**) and quantification (**f**) of BrdU-positive cells in mandibular regions from coronal head sections. At least twenty-five sections were randomly selected from four pairs of E13.5 *Wnt1*^*cre*^*;Tfrc*^*f/f*^ mutants and controls. NS, no significant difference. Scale bar, 0.5 mm (**a**–**d**), 0.1 mm (**e**). BA1, first branchial arch; BA2, second branchial arch; FNP, frontonasal prominence

To explore the possible cellular mechanism by which Tfrc controls mandibular development, we first examine whether Tfrc regulates CNCCs migration. Whole-mount AP-2*α* staining in E9.5 and E10.5 embryos was performed to track the migratory CNCCs, and the results showed comparable expression level and distribution pattern of AP-2*α* in *Wnt1*^*cre*^*;Tfrc*^*f/f*^ mutants and control littermates (Figures 4a and b). To confirm this result, *Wnt1*^*cre*^*;Tfrc*^*f/+*^ mice were crossed with *Tfrc*^*f/f*^;*Rosa26*-reporter mice to get the *Wnt1*^*cre*^*;Tfrc*^*f/f*^*;R26R* mutants. As expected, *Wnt1*^*cre*^*;Tfrc*^*f/f*^*;R26R* mutants also showed mandibular hypoplasia and cleft palate (data not shown). Whole-mount X-gal staining revealed normal CNCCs migration and distribution to FNP, BA1 and BA2 in *Wnt1*^*cre*^*;Tfrc*^*f/f*^*;R26R* mutants as compared with controls (Figures 4c and d). Next, we examined whether cell proliferation was affected by the loss of *Tfrc*. The NCC-derived mesenchymal cell proliferation rate within mandibular regions, as measured by BrdU (5-bromo-2′-deoxy-uridine) incorporation, was similar between E13.5 *Wnt1-cre; Tfrc*^*f/f*^ mutants and controls (Figures 4e and f). Therefore, these results indicate that *Tfrc* ablation in NCCs does not affect CNCCs migration or proliferation.

### *Tfrc* is required for osteochondrogenic differentiation

**Figure 5:**
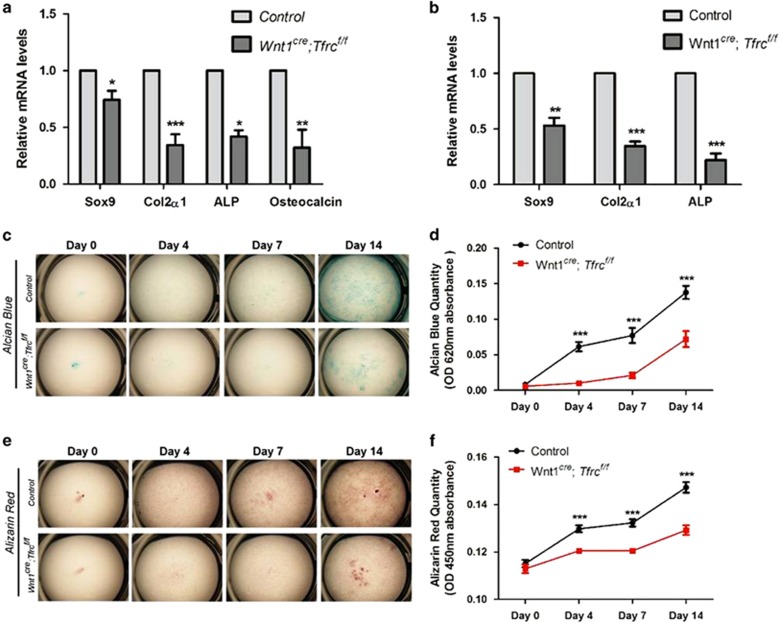
Impaired osteochondrogenic differentiation in *Wnt1*^*cre*^*;Tfrc*^*f/f*^ mutants. (**a**) Expression levels of chondrocyte and osteoblast genes in mandible tissue isolated from E13.5 *Wnt1*^*cre*^*;Tfrc*^*f/f*^ mutants and controls. Data shown are normalized ratio of *Wnt1*^*cre*^*;Tfrc*^*f/f*^ /Control (mean±S.E.M); student's *t*-test; *P<0.05; **P<0.01; ***P<0.001; *n*≥3. (**b**) Expression levels of chondrocyte and osteoblast genes in cultured primary mandible mesenchymal cells. Mandible mesenchymal cells were cultured and induced to undergo osteochondrogenic differentiation then collected samples at day 7. Data shown are normalized ratio of *Wnt1*^*cre*^*;Tfrc*^*f/f*^/Control (mean±S.E.M); student's *t*-test; **P<0.01; ***P<0.001; *n*=5. (**c**–**f**) Alcian blue (**c**) and Alizarin red (**e**) staining show reduced *in vitro* osteochondrogenic differentiation in *Tfrc* KO cells. Mandible mesenchymal cells were cultured and induced to undergo osteochondrogenic differentiation, then collected and stained with Alcian blue at day 0, 4, 7 and 14. (**d**) and (**f**) quantification of OD 620 nm and OD 450 nm absorbance data from (**c** and **e**), respectively. Data shown are mean±S.E.M; student's *t*-test; ***P<0.001; control, *n*=23; *Wnt1*^*cre*^*;Tfrc*^*f/f*^, *n*=14

To investigate whether the mandibular cartilage and bone defects in *Wnt1*^*cre*^*;Tfrc*^*f/f*^ mutants were due to abnormal osteochondrogenic differentiation, we analyzed the expression of marker genes that are required for osteochondrogenic differentiation in E13.5 mandibular tissues by RT-qPCR. The mRNA levels of chondrocyte genes *Sox9* and *Col2a1*, and osteoblast gene *ALP* and osteocalcin were all decreased in *Wnt1*^*cre*^*;Tfrc*^*f/f*^ mutants (Figure 5a), suggesting an osteochondrogenesis defect in *Wnt1*^*cre*^*;Tfrc*^*f/f*^ mandible. To examine whether Tfrc directly regulates osteochondrogenic differentiation, *in vitro* osteochondrogenic differentiation assay was performed using mesenchymal cells isolated from control and Tfrc mutant mandible. On induction of differentiation in medium with *β*-glycerophosphate and ascorbic acid, control cells showed time-dependent cartilage matrix depositions and mineralized matrix depositions (Figures 5c–f). In contrast, these depositions were reduced in *Tfrc* KO cells (Figures 5c–f), suggesting a requirement of Tfrc for osteochondrogenic differentiation. Moreover, expression of *Sox9*, *Col2a1* and *ALP* was decreased in *Tfrc* KO cells (Figure 5b), which further supports for a differentiation defect induced by *Tfrc* deletion. Therefore, these results indicate that Tfrc is required for chondrogenic and osteogenic cell differentiation.

### *Tfrc* deletion suppressed TGF-*β* and BMP signaling in mandible

**Figure 6:**
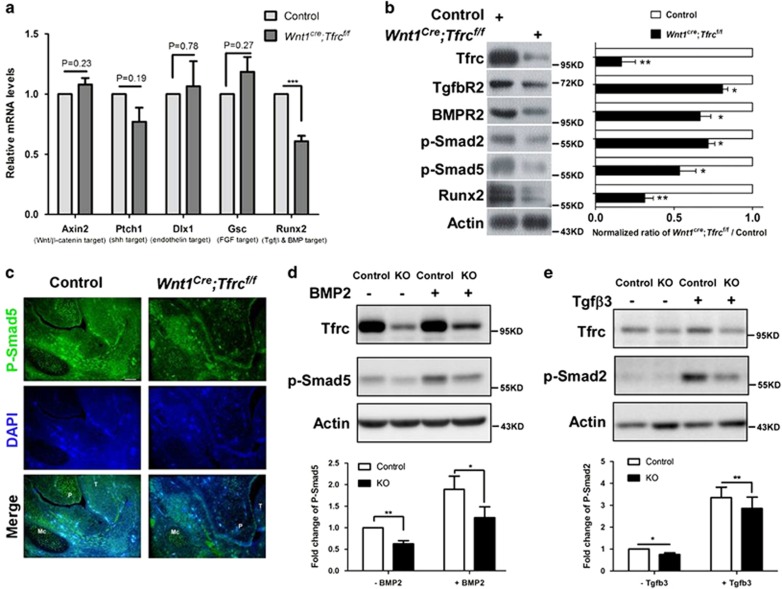
Loss of *Tfrc* in NCCs suppresses the activation of TGF-*β* and BMP signaling. (**a**) Expression levels of indicated signaling pathways targeted genes in mandible tissues dissected from E13.5 *Wnt1*^*cre*^*;Tfrc*^*f/f*^ mutants and controls. Data shown are normalized ratio of *Wnt1*^*cre*^*;Tfrc*^*f/f*^ /Control (mean±S.E.M); student's *t*-test; NS, no significant difference; ***P<0.001; *n*≥3. (**b**) Expression levels of proteins involved in TGF-*β* and BMP signaling in E13.5 mandible tissues. Data shown are normalized ratio of *Wnt1*^*cre*^*;Tfrc*^*f/f*^ /Control (mean±S.E.M); student's *t*-test; *P<0.05; **P<0.01; *n*≥3. (**c**) Immunostaining of P-Smad5 in E13.5 mandible sections. P, palate; T, tongue; Mc, Meckel's cartilage. Scale bar, 100 *μ*m. (**d** and **e**) Immunoblotting and quantification of P-smad5 (**c**) and P-smad2 (**d**) in cultured primary mandibular mesenchymal cells. Mandibular mesenchymal cells were cultured and induced to undergo osteochondrogenic differentiation for 7 days, then treated with BMP2 (100 ng/ml) or Tgf*β*3 (50 ng/ml) for 30 min at 37 °C. Data shown are normalized ratio of KO/Control (mean±S.E.M); student's *t*-test; *P<0.05; **P<0.01; *n*=3

A couple of signaling pathways including Wnt/*β*-catenin, Sonic hedgehog (Shh), endothelin, fibroblast growth factor (FGF) family and TGF-*β* superfamily have been implicated as critical regulators for craniofacial development. We then tested whether Tfrc regulates craniofacial development through these signaling pathways. *Axin2*, *Ptch1*, *Dlx1*, *Gsc* and *Runx2*, which are, respectively, typical downstream target of the indicated five pathways, were selected for expression analysis by using RT-qPCR. As shown, only *Runx2*, a target gene of TGF-*β* superfamily signaling, was downregulated in *Wnt1*^*cre*^*;Tfrc*^*f/f*^ mutants (Figure 6a). Protein expression levels of TGF-*β*R2, BMPR2 and Runx2 were all decreased in *Wnt1*^*cre*^*;Tfrc*^*f/f*^ mutants mandibular tissue; moreover, phosphorylation of smad2 and smad5 (P-samd5 and P-smad2), which, respectively, represents the activation of TGF-*β* and BMP signaling, were also hugely reduced (Figures 6b and c), suggesting that Tfrc is required for the activation of TGF-*β* and BMP signaling. Next, we confirmed this result in cultured mandibular mesenchymal cells. Results showed that *Tfrc* deletion dramatically suppressed the phosphorylation of smad5 and smad2 induced by TGF-*β* and BMP2 treatment (Figures 6d and e). Combined, these results suggest that Tfrc is critical for the activation of TGF-*β* and BMP signaling, and Tfrc-dependent activation of these signaling pathways likely contributes to craniofacial morphogenesis by controlling mandibular development.

## Discussion

In this study, we investigated the function of Tfrc in NCCs by using a *Wnt1*^*cre*^-LoxP system and found a previously unknown role of Tfrc during craniofacial development. Specific deletion of *Tfrc* in NCCs leads to craniofacial defects including cleft palate, severe respiratory distress and inability to suckle, resembling the human PRS (OMIM 261800). Consequently, *Wnt1*^*cre*^*;Tfrc*^*f/f*^ mutant pups die within 24 h after birth. Further examinations indicate that mandible hypoplasia resulted from Meckel's cartilage development defects is presumably a primary cause for cleft palate in *Wnt1*^*cre*^*;Tfrc*^*f/f*^ mutants. Thus, our study provides evidence that Tfrc-mediated signaling in NCCs is crucial for craniofacial development.

*Wnt1*^*cre*^*;Tfrc*^*f/f*^ mutant mice simultaneously exhibit the cleft palate and hypoplastic mandible phenotypes. Timing histological and anatomical analysis revealed that the cleft palate in *Wnt1*^*cre*^*;Tfrc*^*f/f*^ mutants is directly caused by failed elevation of palatal shelves, while hypoplastic mandible resulted from retarded growth of Meckel's cartilage. However, the lack of either cleft palate or hypoplastic mandible in *Nestin-Cre*-mediated *Tfrc* KO mice suggests a different mechanism. Considering that *Wnt1*-cre-driven deletion of *Tfrc* occurs in mandible and palatal mesenchyme, while *Nestin-cre*-mediated *Tfrc* ablation mainly happens in the mesenchyme and epithelium of palatal shelves but not in mandible, we propose that cleft palate in *Wnt1*^*cre*^*;Tfrc*^*f/f*^ mutant is a second consequence due to mandibular development defects. Mechanistically, *Tfrc* KO in NCCs primarily reduce the extension of Meckel's cartilage that is required for correct forward growth and elongation of lower jaw, then consequently cause mandibular hypoplasia. As a second result, defective mandibular maturation restricts the elevation of palatal shelves from vertical to horizontal position and then leads to cleft palate in *Wnt1*^*cre*^*;Tfrc*^*f/f*^ mutants. Our finding is consistent to previous reports that obstruction of mandibular growth leads to cleft palate in several mutants, such as *Hoxa2*^−/−^,^17^ *Egfr*^−/−^,^18^ *Dmm*,^7^ *Wnt1*^*Cre*^*;Alk2*^*flox/flox*^ mutants,^19^ *Snai1*^+/–^;*Snai2*^−/−,^^20^ *csp1, Prdm16*^*Gt683Lex*^ mutants ^21^ and *Ptprs*^−/−^;*Ptprf*^−/−^.^22^ Interestingly, cleft palate in human PRS is also due to the failure of tongue translocation or mandibular growth defects, suggesting that *Wnt1*^*cre*^*;Tfrc*^*f/f*^ mutant mice not only morphologically but also mechanistically resemble human PRS.

What is the cellular mechanism for mandibular defects in *Wnt1*^*cre*^*;Tfrc*^*f/f*^ mutant? Fine control of NCC migration, proliferation and differentiation is essential for Meckel's cartilage processing and mandible elongation. Our analysis revealed that Tfrc is not required for NCCs migration or proliferation. However, the differentiation of chondrogenic and osteogenic cell within mandible are severely suppressed by *Tfrc* deletion (Figure 5). Further molecular analysis showed that the levels of *Sox9*, *Col2a1*, *ALP* and *Osteocalcin*, which are highly expressed within differentiated cells, are decreased in *Tfrc* KO cells (Figures 5a and b), confirming that the differentiation process is delayed by *Tfrc* ablation. Thus, these results indicate that the hypoplastic mandible in *Wnt1*^*cre*^*;Tfrc*^*f/f*^ mutant is likely a result of impaired chondrogenic and osteogenic differentiation.

What is the molecular mechanism for mandibular defects in *Wnt1*^*cre*^*;Tfrc*^*f/f*^ mutant? TGF-*β* superfamily signaling is known to perform essential roles in mandible and Meckel's cartilage development. Disruption of TGF-*β* superfamily signaling by either complete or conditional inactivation of *Bmp4*, *TGF-β*2, *TGF-βR*2, *Alk*5, *Alk*2 or *Smad*4 results in abnormal development of Meckel's cartilage and mandible, which lead to cleft palate.^19, 32, 33, 34, 35, 36, 37^ In the present study, we found that *Tfrc* deletion in NCCs dramatically suppresses both TGF-*β* and BMP signaling. In consistence, *Tfrc* KO mice show cleft palate and hypoplastic mandible that are similar to the phenotypes observed in mice lacking TGF-*β* or BMP signaling. Our data thus suggest that Tfrc is a critical regulator for TGF-*β* superfamily signaling activation during craniofacial development.

How does Tfrc control TGF-*β* superfamily signaling? Here, we speculate that Tfrc may control the endocytosis of TGF-*β* and BMP receptors and is then required for TGF-*β* and BMP activation. First, TGF-*β* and BMP signaling activation is dependent on clathrin-mediated endocytosis of TGF-*β* and BMP receptors.^38, 39, 40^ Second, adaptor protein 2 (AP-2), an essential adaptor for clathrin-mediated endocytosis, is identified to interact with TGF-*β* and BMP receptors.^41, 42^ Third, local clustering of Tfrc at cell surface promotes clathrin-coated pits initiation and clathrin lattices formation,^43, 44, 45^ and a tight association between AP-2 and Tfrc was detected in our study (data not shown) and in previous literature.^46^ Fourth, *Tfrc* deletion in NCCs suppresses both TGF-*β* and BMP signaling. So, it is plausible that Tfrc functions as a facilitator to control TGF-*β* and BMP receptor endocytosis via binding clathrin adaptor AP-2. Of note, *Tfrc* deletion may also affect caveolin-dependent endocytosis of TGF-*β* and BMP receptors. It has been documented that TGF-*β* and BMP receptors are internalized into cytoplasm through two distinct endocytic pathways, clathrin-mediated endocytosis and caveolin-dependent endocytosis. Clathrin-mediated endocytosis is important for the signaling activation, whereas caveolin-dependent endocytosis is required for receptor degradation.^38, 39, 40^ We indeed found that *Tfrc* deletion strikingly decreased the protein level of both TGF-*β* and BMP receptors, indicating increased degradation of TGF-*β* and BMP receptors in response to *Tfrc* deletion. We hypothesize that this effect is possibly due to accelerated caveolin-dependent endocytosis of TGF-*β* and BMP receptors in *Tfrc* KO cells. Likely, *Tfrc* deletion switches the internalization pathway of TGF-*β* and BMP receptors from clathrin-mediated to caveolin-mediated endocytosis and thereby leads more receptors to be destined for degradation. Certainly, it is also possible that Tfrc negatively regulates caveolin-dependent endocytosis of TGF-*β* and BMP receptors in a direct manner. Further experiments will be needed to investigate these possible mechanisms in detail.

## Materials and Methods

### Generation of *Wnt1*^*cre*^*;Tfrc*^*f/f*^ mutant mice and genotyping

All mice were kept in specific pathogen-free standard condition and all experiments were conducted in accordance with the guidelines and under the approval of Animal Care Committees at Sichuan University. The generation of *Wnt1*^*cre*^*;Tfrc*^*f/f*^ mutants were obtained by cross-mating of *Tfrc*^flox/flox^ mice (from Andrews)^29^ with heterozygote *Wnt1*^*cre*^*; Tfrc*^*f/+*^. *Wnt1*^*cre*^*; Tfrc*^*f/+*^heterozygous had no obvious phenotype, control littermates were taken as *Tfrc*^*f/+*^, *Tfrc*^*f/f*^ or *Wnt1*^*cre*^*; Tfrc*^*f/+*^. Genotyping was performed by PCR using primers listed in Supplementary Material and Supplementary Table S2.

### Skeletal preparations

Embryonic or neonatal controls and *Wnt1*^*cre*^*;Tfrc*^*f/f*^ mutants were de-skinned, eviscerated and fixed in 100% ethanol for 24 h, then followed by 100% acetone to remove fat. Skeletons were stained in Alcian blue solution (150 mg/l Alcian blue, 80% ethanol and 20% acetic acid) for 48 h at 37 °C, then washed in 95% ethanol for 8-12 h and cleared in 2% KOH solution for 24 h, followed by staining overnight in Alizarin red solution (50 mg/l Alizarin red in 2% KOH). Finally, the skeletons were cleared in water and stored in 20% glycerol.

### Histological staining

For immunostaining, antibodies against Tfrc and AP-2*α* were purchased from Invitrogen (136800) and Epitomics (3154-1) and used at 1 : 400.

Whole-mount immunohistochemistry was performed as previously described.^47^ AP-2*α* antibody was used at 1 : 100.

Whole-mount X-gal staining was performed using the *In Situ* *β*-galactosidase Staining Kit (Beyotime, Haimen, Jiangsu, China, RG0039), following the manufacturer's recommendations.

For histological examination, embryos and neonatal mice were fixed in 4% paraformaldehyde overnight at 4 °C, then dehydrated in gradient sucrose solution and embedded in OCT, and cryosectioned at 14 *μ*m. Sections were stained with hematoxylin and eosin following standard procedures.

### Cell proliferation assay

Cell proliferation was evaluated by DNA synthesis activity through intraperitoneal BrdU injection (50 *μ*g/g body weight) for 1.5 h, then mice were killed and embryos were fixed in 4% paraformaldehyde. Sections were treated with 2 N HCl at 37 °C for 20 min then neutralized with 0.1 M sodium borate for 10 min. BrdU antibody (Santa Cruz, Dallas, TX, USA, sc32323, 1 : 200) was used to detect proliferative cells.

### Palatal shelves culture

Palatal shelves culture was performed as previously described with minor modification.^33, 48^ Briefly, palatal shelves were dissected from E13.5 embryos and placed on Millipore filters as the two segments just touching at the medial edge, cultured for 3 days and fixed in 4% paraformaldehyde followed by histological hematoxylin and eosin analysis.

### Immunoblotting analysis

Antibodies used in immunoblotting analysis: Tfrc (Invitrogen, Waltham, MA, USA, 136800), Phospho-Smad5 (Epitomics, Burlingame, CA, USA, 2224-1), Phospho-Smad2 (Cell Signaling Technology, 3101), BMPR2 (Epitomics, S0778), Tgf*β*R2 (Cell Signaling Technology, Burlingame, CA, USA, 3713), Runx2 (Millipore, Merck, KGaA, Darmstadt, Germany, 05-1478), *β*-actin (Santa Cruz, sc-47778). The immunoblotting analysis followed standard procedures.

### Mandibular mesenchymal cell culture

Primary mandibular mesenchymal cell culture and osteochondrogenic differentiation were performed as previously described with minor modification.^49, 50^ Briefly, mandible from E13.5 embryo was dissected and incubated with 0.25% trypsin at 37 °C for 30 min. Isolated cells were plated at a density of 3 × 10^4^ cells/cm^2^ and cultured in complete medium (DMEM containing 10% FBS supplemented with penicillin, streptomycin, l-glutamate and sodium pyruvate). After 24 h (day 0), culture medium was supplemented with 10 mM *β*-glycerophosphate, 50 *μ*g/ml ascorbic acid and 2.5 *μ*M retinoic acid to induce osteochondrogenic differentiation. Followed by indicating days, cells were fixed then stained with Alcian blue and Alizarin red to evaluate cell differentiation, the staining and quantification method were performed as previously described.^50, 51^

### Real-time quantitative PCR

Total RNA was extracted using the RNeasy kit (QIAGEN, Hilden, Germany, 74134), and cDNA was synthesized with the TransScript One-Step gDNA Removal and cDNA Synthesis SuperMix (Transgen, Beijing, China, AT311). Quantitative real-time PCR was performed using SYBR Green PCR Master Mix (Applied Biosystems, Waltham, MA, USA, 4367659). Primer sequences used for qPCR were listed in Supplementary Material and Supplementary Table S2, according to previously published results.^22, 52^ Expression levels of mRNA were normalized to *β*-actin.

### Statistical analysis

Statistical analyses were performed using two-tailed Student's *t*-test. Data are presented as mean±S.E.M; *P*-value<0.05 indicated significant difference.

## Abbreviations

AP-2: adaptor protein 2
BA: branchial arch
BMP: bone morphogenetic protein
BrdU: 5-bromo-2′-deoxy-uridine
CNCCs: cranial neural crest cells
E: embryonic day
FNP: frontonasal prominence
KO: knockout
MEE: medial edge epithelium
NCCs: neural crest cells
P: postnatal day 0
Ppmx: palatal process of maxilla
Pppl: palatal process of palatine
PRS: Pierre Robin Sequence
Tfrc: transferrin receptor
TGF-*β*: transforming growth factor-*β*
