# Supplementary figures and images for: Transferrin receptor facilitates TGF-β and BMP signaling activation to control craniofacial morphogenesis
Source: Cell Death Dis. 2016 Jun 30;7(6):e2282–. doi: 10.1038/cddis.2016.170 (PMC5108332; doi:10.1038/cddis.2016.170)

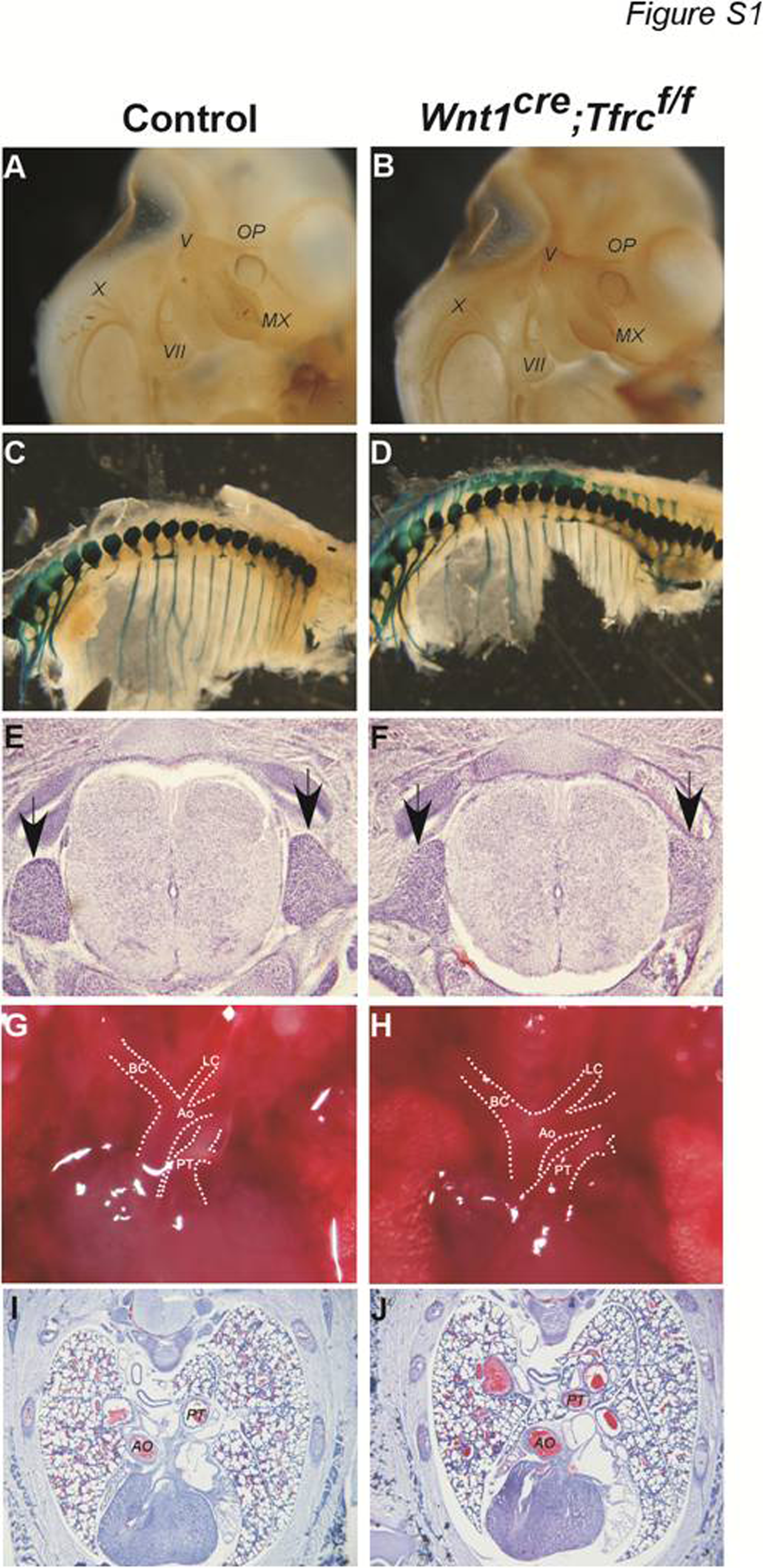

Supplement: Supplementary Figure 1 [file cddis2016170x1.tif]

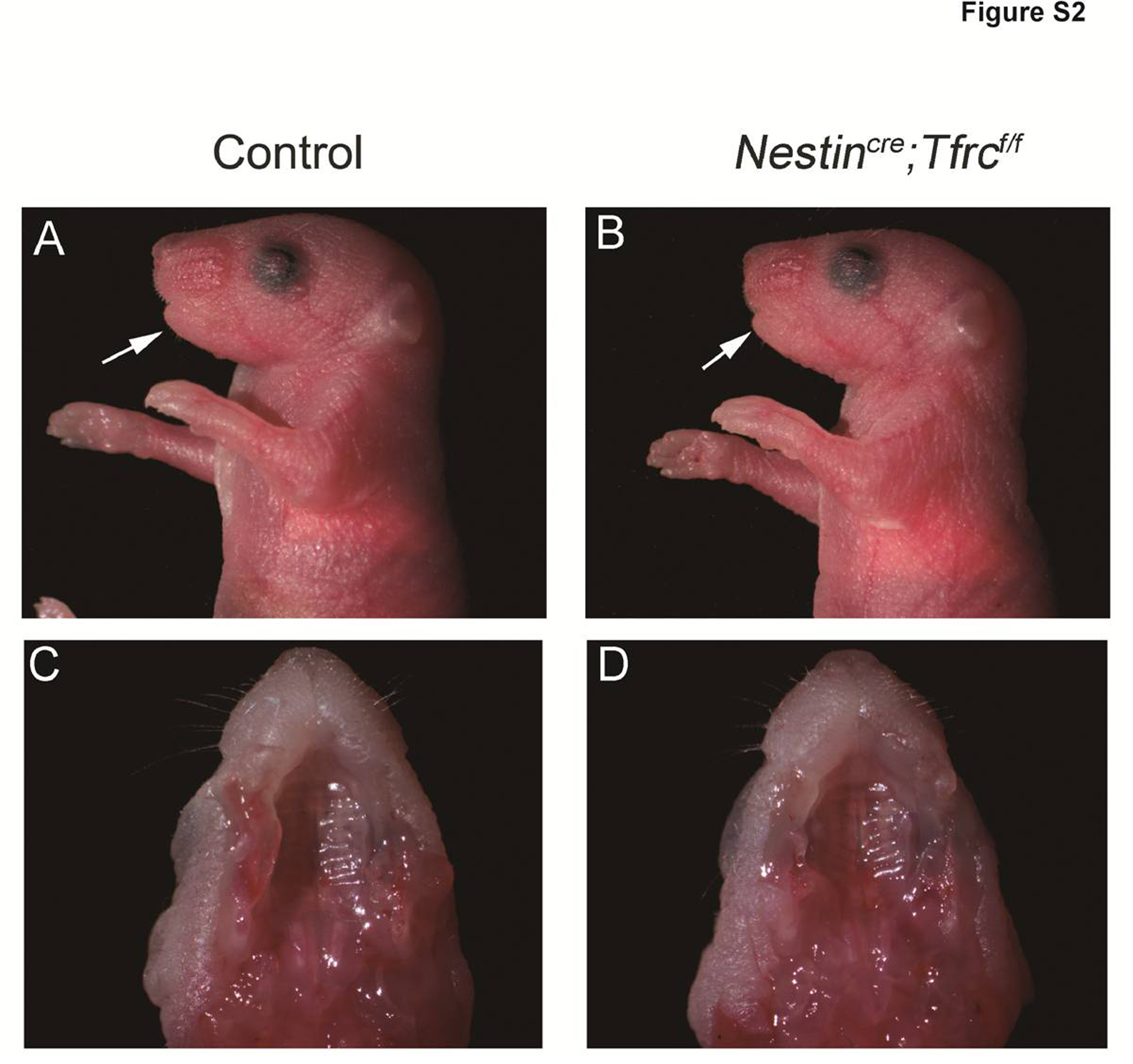

Supplement: Supplementary Figure 2 [file cddis2016170x2.tif]
