# Supplementary material for: Transferrin receptor facilitates TGF-β and BMP signaling activation to control craniofacial morphogenesis
Source: Cell Death Dis. 2016 Jun 30;7(6):e2282–. doi: 10.1038/cddis.2016.170 (PMC5108332; doi:10.1038/cddis.2016.170)
Supplement: Supplementary Information [file cddis2016170x3.doc]

**Figure S1. Gross morphologies of craniofacial nerve, dorsal root ganglion and cardiac outflow tract are normal in *Wnt1^cre^;Tfrc^f/f^* mutants.**

(A, B) Whole-mount staining of anti-neurofilament marker 2H3 in E9.5 *Wnt1^cre^;Tfrc^f/f^* mutant and control.

(C, D) The lateral view of whole-mount X-gal staining of dorsal root ganglions in E14.5 *Wnt1^cre^;Tfrc^f/f^* mutant and control.

(E, F) Hematoxylin and eosin staining of E18.5 body cross sections, arrows indicate dorsal root ganglions.

(G, H) Photos of heart in P0 *Wnt1^cre^;Tfrc^f/f^* mutant and control. Dotted lines indicate the cardiac outflow tract.

(I, J) Hematoxylin and eosin staining of body cross sections at P0.

V, trigeminal ganglion; VII, facial ganglion; X, vagus ganglion; OP, ophthalmic nerve; MX, maxillary nerve; AO, aorta; BC, brachiocephalic artery; LC, left carotid; PT, pulmonary trunk.

**Figure S2. Gross craniofacial morphogenesis is normal in *Nestin^cre^;Tfrc^f/f^* mutant.**

(A, B) The lateral view of newborn mice. Arrows indicate mandible in *Nestin^cre^;Tfrc^f/f^* mutant and control.

(C, D) The ventral view of oral cavity. Palatal shelves fused normally in newborn *Nestin^cre^;Tfrc^f/f^* mutant and control.

**Table S1**

| Table S1. Abnormalities in branchial arch-derived structures at P0 *Wnt1^cre^;Tfrc^f/f^* | | |
| --- | --- | --- |
| **Arch** | **Structure** | **Abnormality** |
| 1 | Palatine bones | Failure of elevation and fusion |
|  | Palatal process of maxilla | Failure of fusion |
|  | Tympanic ring bones | Deformed and weakened |
|  | Mandible | Shortened and mis-angled |
|  | Coronoid process | Hypoplasia |
|  | Condylar process | Hypoplasia |
| 2 | Lesser horns of the hyoid | Fusion with hyoid |

**Table S2**

| Gene | Use | Forward primer | Reverse primer |
| --- | --- | --- | --- |
| *Tfrc* | PCR | CAGTAATCCCAGAGGAATCATTAG | CTAAACCGGGTGTATGACAATG |
| *Wnt1^cre^* | PCR | CATACCTGGAAAATGCTTCTGTCC | TCCCCAGAAATGCCAGATTACG |
| *Nestin^cre^* | PCR | CCGCTTCCGCTGGGTCACTGT | 1.CTGAGCAGCTGGTTCTGCTCCT 2.CTGAGCAGCTGGTTCTGCTCCT |
| *Axin2* | qPCR | CCATTTTGGACGACCACCTCTC | GAAGAAGGGTATGACACTGCTGATG |
| *Ptch1* | qPCR | TCTGCTGGGTGTACTGATGC | TCAGGACACGGTCCAAAGA |
| *Dlx1* | qPCR | TTCATCTGACGCTGAGTGTTGG | TTTCCTGTCCTTGTTCCCTCTTC |
| *Gsc* | qPCR | TAAGAACCGCCGAGCCAAG | CCGAGTCCAAATCGCTTTTACC |
| *Sox9* | qPCR | TCGGAACTGCCTGGAAACTTC | GAGGGAGGGAAAACAGAGAACG |
| *Col2a1* | qPCR | GAGCAGCAAGAGCAAGGAAAAG | CAGTGGACAGTAGACGGAGGAAAG |
| *ALP* | qPCR | CACGCGATGCAACACCACTCAGG | GCATGTCCCCGGGCTCAAAGA |
| *Runx2* | qPCR | ACCAGTCTTACCCCTCCTATCTGAG | GCAGTGTCATCATCTGAAATACGC |
| *Osteocalcin* | qPCR | CTCACAGATGCCAAGCCCA | CCAAGGTAGCGCCGGAGTCT |
| *β-actin* | qPCR | TGTGGTGGTGAAGCTGTAGC | GACGACATGGAGAAGATCTGG |
